# Protective Effects of Curcumin on the Outcome of Cryopreservation in Human Sperm

**DOI:** 10.1007/s43032-021-00572-9

**Published:** 2021-04-16

**Authors:** Marianna Santonastaso, Filomena Mottola, Concetta Iovine, Nicola Colacurci, Lucia Rocco

**Affiliations:** 1 Department of Woman, Child and General and Special Surgery, University of Campania “Luigi Vanvitelli”, Napoli, Italy; 2 Department of Environmental, Biological and Pharmaceutical Sciences and Technologies, University of Campania “Luigi Vanvitelli”, Caserta, Italy

**Keywords:** Sperm quality, Curcumin, Sperm cryopreservation, Glutathione peroxidase 4, Oxidative stress, Sperm DNA fragmentation

## Abstract

Cryopreservation causes decreased sperm fertility potential due to reactive oxygen species (ROS) production and physical-chemical damage, resulting in reduced sperm viability and motility. The addition of antioxidants to freezing media could protect sperm from cryo-damage, counteracting the harmful effects of ROS. The aim of this study was to assess the effects of curcumin supplementation in freezing medium on preventing cryo-damage in human semen. Semen samples collected from fertile men were cryopreserved in freezing medium supplemented with different concentrations of curcumin (2.5, 5, 10, and 20 μM). After freezing-thawing, sperm parameters, DNA fragmentation, intracellular ROS, and glutathione peroxidase 4 (*GPX*4) gene expression were evaluated. Supplementation with 20 μM curcumin in freezing medium caused increases in progressive and nonprogressive motility and significant reductions in intracellular ROS and DNA fragmentation in frozen-thawed sperm cells. Following cryopreservation, *GPX*4 mRNA expression was significantly upregulated in thawed semen supplemented with 20 μM curcumin compared to the control. The results showed that curcumin supplementation in freezing medium was protective against human sperm parameters and sperm DNA, counteracting oxidative damage induced by the freeze-thaw process.

## Introduction

Human sperm cryopreservation is a widely used practice in assisted reproductive technology (ART) centres for various reasons, such as fertility preservation before cancer treatment [1]; spermatozoa storage to overcome oligozoospermic and azoospermic conditions, percutaneous epididymal sperm aspiration (PESA) or testicular sperm extraction (TESE) due to ejaculatory dysfunction or spinal cord injury; or sperm donation [2].

Freezing and thawing procedures have adverse effects on sperm structure and function, reducing sperm viability, motility and longevity in the female genital tract and consequently decreasing fertility potential [3]. Throughout the freezing and warming processes, spermatozoa are exposed to physical and chemical stresses that result in excessive dehydration, plasma membrane disintegration, acrosome leakage, mitochondrial injury, metabolic-functional changes, and DNA fragmentation [4–8]. This sperm cryo-damage is mediated by reactive oxygen species (ROS) production [9–11]. ROS can alter the sperm antioxidant defence system, resulting in lipid peroxidation, membrane fluidity reduction, membrane enzyme and ion channel inactivity, decreased sperm motility, increased apoptosis, sperm-oocyte fusion defects, early miscarriage and embryonic genetic mutations [12, 13].

Antioxidants are molecules that are able to inhibit or reduce oxidative processes by scavenging released free radicals [14]. Several antioxidants have been suggested to be helpful in male infertility treatment to counteract ROS and preserve sperm motility, viability and functionality, such as vitamins E and C, as well as selenium (Se), zinc (Zn) [15, 16] and ellagic acid [17]. Moreover, antioxidants have shown positive effects on sperm cryopreservation; the addition of vitamin E in freezing medium significantly reduces sperm ROS levels following the thawing process [18].

For this reason, enriching culture freezing systems with antioxidants could be an effective approach to counteract sperm damage induced by cryopreservation [19–27].

Curcumin, a type of antioxidant, is a yellow phenolic pigment that is a component of the rhizome of *Curcuma longa* with a broad range of biological and pharmacological functions, such as anti-inflammatory, antineoplastic, antioxidant and anti-mutagenic activities [28–30]. The safety of curcumin was reported by the United States Food and Drug Administration [31]. Curcumin presents a dual mechanism of action as an antioxidant; one mechanism is due to its chemical structure, and the other is linked to its ability to stimulate the production of antioxidant enzymes. Curcumin contains different functional antioxidant groups, such as β-diketo groups, carbon-carbon double bonds and phenyl rings [32]. The antioxidant activity of curcumin was also associated with its phenolic and/or central methylenic groups [33, 34], while its chelating activity and ability to capture ferrous ions are due to functional carbonyl groups [32]. Moreover, curcumin exhibits antioxidant activity by regulating transcription factors and antioxidant enzymes, such as haem oxygenase-1 (HO-1) and nuclear factor erythroid 2-related factor 2 (Nrf2), upregulating common antioxidative activities (superoxide dismutase (SOD) and glutathione (GSH)) and inhibiting cytokine production (e.g. interleukin-1β (IL-1β), tumour necrosis factor-α (TNF-α) and interleukin 12 (IL-12)) [35–37].

In sperm cells, curcumin improved capacitation, acrosome reaction and fertilization in vitro and in vivo [38–40]. This antioxidant could increase sperm motility in patients with leucocytospermia and improve semen parameters in asthenoteratospermia by regulating the levels of the transcription nuclear factor Nrf2 [41].

Curcumin has a protective effect against spermatogenesis defects induced by titanium dioxide nanoparticles (n-TiO_2_) [42] and restores testicular damage induced by alcohol, cisplatin, aflatoxin, metronidazole, ischaemia reperfusion and cadmium exposure in mice [43–48]. It also improves the histopathological alterations induced by monosodium glutamate in the testis and epididymis, increasing sperm count in rats [49].

The positive effects of curcumin supplementation on sperm freezing media have been reported in various animal models, such as Angora goats, bulls, Wistar mice and buffalos [21, 50–52]. It has been reported that curcumin could improve bull spermatozoa and rat testes subjected to induced oxidative stress and could improve cryopreserved boar spermatozoa, increasing progressive motility and acrosome integrity [53–55].

In rams, curcumin had protective effects on frozen-thawed sperm parameters at different doses; antioxidant supplementation resulted in a higher percentage of sperm acrosome integrity and provided strong protection in terms of sperm mitochondrial activity in comparison to the control [56].

These data have led to the hypothesis that curcumin may have an effect on preventing human sperm damage induced by cryopreservation, so the aim of this study was to test the effects of curcumin in preventing cryo-damage during the freeze/thaw process of sperm from fertile men. To date, no studies concerning this property have been carried out on human spermatozoa. We evaluated the effects of different concentrations (2.5, 5, 10 and 20 μM) of curcumin supplementation in freezing medium on human sperm parameters, intracellular ROS, DNA fragmentation and glutathione peroxidase 4 (*GPX*4) antioxidant gene expression after freeze-thaw cycling. Glutathione peroxidase (*GPX4*), which encodes the glutathione peroxidase 4 (Gpx4) protein, protects cells from oxidative stress caused by cell membrane peroxidation. Gpx4 is important for normal spermatozoa development, as it protects sperm cells from oxidative stress and is a necessary structural protein in mature spermatozoa, and Gpx4 expression alterations are associated with male infertility [57, 58].

## Materials and Methods

### Chemicals

Curcumin powder was supplied by Sigma-Aldrich (CAS number 458-37-7). Curcumin stock solution (0.1 M) was prepared using 96% ethanol as a solvent.

### Sample Collection and Analysis

The subjects were recruited from our Reproduction Biology Laboratory (University of Campania “Luigi Vanvitelli”). We did not require approval from the Ethics Committee because the study involved neither therapeutic interventions nor any change to our routine sperm analysis; moreover, written informed consent was obtained from each subject before their inclusion in the study. Subjects with any history of drug addiction, smoking or alcohol consumption, prolonged diseases such as varicocele or drug consumption, including antioxidant consumption, were not included. The ejaculates were collected from men between 30 and 42 years old (36.5 ± 6.2 years old mean age) by masturbation after 3–5 days of recommended abstinence. After liquefaction at room temperature for 30 minutes (min), the semen volume, sperm concentration, viability, motility and morphology were determined according to the 2010 WHO guidelines [59]. Sperm motility was evaluated by optical microscopy at 400× magnification and was classified according to WHO guidelines 2010 as progressive, nonprogressive or immotile. Sperm viability was examined using eosin–nigrosin staining, while sperm morphology was evaluated on Testsimplets® (ORIGIO, Italia) prestained slides. For the study, we selected 60 ejaculates with good semen parameters according to the 2010 WHO guidelines (Table 1).

**Table 1:** Parameters of semen selected for the study (*n* = 60). Sperm parameters were expressed as mean ± SD

Sperm parameters	Mean ± SD
Semen volume (mL)	3.16 ± 1.43
Sperm concentration (× 10^6^ sperm/mL)	65.36 ± 23.94
Motility (%)	
Progressive	49.67 ± 4.66
Non-progressive	27.0 ± 8.51
Immotile	23.33 ± 11.44
Normal morphology (%)	24.42 ± 8.62
Viability (%)	78.75 ± 10.18

### Exposure Procedure

The pooled samples (*n* = 60) were divided into five aliquots (10 × 10^6^ sperm/mL) (*n* = 12 for each group) and frozen in the presence of freezing medium plus different concentrations of curcumin: 0 (vehicle control group), 2.5 μM, 5 μM, 10 μM and 20 μM. In the vehicle control group, ethanol was added at the same concentration used to dissolve curcumin in each group. We added 3.4 μL of curcumin stock solution to 1.7 mL of sample/freezing medium mixture in the 20 μM curcumin group; 1.7 μL of curcumin stock solution to the sample/freezing medium mixture in the 10 μM curcumin group; and 0.85 μL and 0.425 of curcumin stock solution to the sample/freezing medium mixture in the 5 μM and 2.5 μM curcumin groups, respectively. The sperm freezing procedures are described below. After 7 days of freezing, followed by thawing, as described below, the samples were analysed for sperm motility, viability, DNA fragmentation, intracellular ROS levels and *GPX*4 gene expression. All experiments were performed in triplicate.

### Sperm Freezing and Thawing

The sperm freezing and thawing procedure was conducted according to the manufacturer’s instructions (SpermFreeze, FertiPro N.V., Beernem, Belgium). Briefly, 1 mL of sample was diluted dropwise with 0.7 mL of freezing medium (SpermFreeze, FertiproN.V., Beernem, Belgium). The medium is a commercial cryoprotectant consisting of 15% glycerol in 4-(2-hydroxyethyl)-1-piperazine ethanesulfonic acid (HEPES) buffer supplemented with human serum albumin (HSA). After 30 min for the equilibration of the sample/medium mixture at room temperature, the mixture was transferred into cryovials (Thermo Scientific Nunc, Denmark). The cryovials were set into a metal surface under liquid nitrogen vapour for 15 min (slowly frozen) and then immersed in liquid nitrogen (− 196 °C liquid nitrogen) for storage.

After cryostorage for 7 days, the samples were thawed in a tap water container at 37 °C for 4–5 min. Then, the sperm samples were resuspended in sperm washing medium (FertiPro N.V., Beernem, Belgium) and centrifuged at 1500 revolutions per minute (rpm) for 5 min. The sperm pellet was resuspended in sperm washing medium (FertiPro N.V., Beernem, Belgium) and analysed for sperm motility, viability, DNA fragmentation, intracellular ROS levels and *GPX*4 gene expression.

### Intracellular ROS Measurement

Intracellular sperm ROS levels were quantified by a DCF assay with a 2,7-dichlorodihydrofluorescein diacetate (DCFH_2_-DA) probe according to Santonastaso et al. [60]. DCFH_2_-DA (13 μM, Sigma-Aldrich) was added to a 150-μL semen sample and incubated at 37 °C for 30 min in the dark. After washing in 1× phosphate-buffered saline (PBS, Sigma-Aldrich), the sperm cells were counterstained with 4′,6-diamidino-2′-phenylindole dihydrochloride (DAPI, Sigma-Aldrich) solution and analysed under a fluorescence microscope (Nikon Eclipse E-600) equipped with BP 330-380 nm and LP 420 nm filters. Intracellular ROS was visually scored and measured as the percentage of sperm cells exhibiting a response (green cells) to total sperm cells. DCF assay was performed in triplicate.

### Sperm DNA Fragmentation Assessment

DNA fragmentation was determined using an In Situ Cell Death Detection Kit (Roche Diagnostics) according to Santonastaso et al. [61]. Fifteen microlitres of sample was placed on glass slides, fixed in 4% paraformaldehyde for 1 h at room temperature and air dried. After 2 min of incubation in permeabilizing solution (sodium citrate, distilled H_2_O and Triton X-100), the glass slides were washed in 1× PBS and air dried, and then, the TUNEL reaction mixture (5 μL of enzyme terminal deoxynucleotidyl transferase solution and 45 μL of label solution) was placed on the slides. Each slide was incubated for 1 h at 37 °C in a humid chamber, stained with DAPI solution for 5 min and analysed under a fluorescence microscope (Nikon Eclipse E-600) equipped with 330–380 nm BP and 420 nm LP filters. Cells with fragmented DNA emitted green fluorescence, while those with intact DNA emitted blue fluorescence due to DAPI. We counted 300–500 cells per slide, distinguishing those with fragmented DNA (green fluorescence) from those with intact DNA (blue fluorescence). The TUNEL assay was performed in triplicate.

### Total RNA Isolation, cDNA Synthesis and Quantitative Real-Time Polymerase Chain Reaction

An SV Total RNA Isolation System (Promega) kit was used to extract total RNA from sperm cells (2 × 10^6^ spermatozoa), and through the use of the GoScript Reverse Transcription System (Promega), 500 ng of total RNA was reverse transcribed into a 25 μL of first-strand cDNA pool following the instructions provided by the manufacturer.

Quantitative real-time polymerase chain reaction (qPCR-RT) was performed in a total volume of 10 μL using the iTaqTM Universal SYBR® Green One-Step Kit (Bio-Rad) comprising the dsDNA binding fluorophore (SYBR Green), thermostable polymerase, magnesium ions and deoxynucleotide triphosphates (dNTPs). The reaction mixture contained 5 μL of 1× Master Mix, 1 μL of forward primer (50 μM), 1 μL of reverse primer (50 μM) (Table 2) and 80 ng of complementary DNA (cDNA). Human *GPX*4 sequences were obtained from GenBank and designed with Primer3 software. The reactions were carried out in triplicate and run on an iCycler Thermal Cycler w/iQ5 Multicolour Real-Time PCR Detection (Bio-Rad) under the following conditions: 95 °C for 10 min for enzyme activation and DNA denaturation; 40 cycles of 95 °C for 15 s and 55 °C for 1 min; and finally, 60 °C + 0.5 °C for 10 min. Fluorescence intensities were analysed using the manufacturer’s CFX Manager (Bio-Rad) software. The relative quantification of *GPX*4 mRNA expression detectable in human sperm cells [62, 63] was calculated using the 2^−ΔΔCt^ method and normalized according to the expression of β-actin as a housekeeping gene. The specificity of the products was evaluated by melting curve analysis [64].

**Table 2:** Real-time q-PCR primers. F: forward primer; R: reverse primer; *GPX*4: glutathione peroxidase 4; *ß-ACT*: ß-actin

Gene	Primer sequence (5′ to 3′)
*GPX*4	F: TCA GCA AGA TCT GCG TGA AC R: CCG GAT GCC ATA GTC AGG AT
*ß-ACT*	F: GGA CTT CGA GCA AGA GAT GG R: AGC ACT GTG TTG GCG TAC AG

### Statistical Analysis

All sperm parameters are expressed as the mean ± standard deviation (SD). Differences in the DNA fragmentation index (DFI) and intracellular ROS among the experimental groups were analysed using unpaired Student’s *t* test with the software package GraphPad Prism 6 [65]. The effect was considered significant if the *p* value was ≤ 0.05 with respect to the vehicle control.

## Results

### Sperm Viability and Motility

No significant difference was observed in the percentage of viable sperm after thawing when each curcumin group (2.5 μM, 5 μM, 10 μM and 20 μM) was compared with the vehicle control group. Our results showed that total motility (progressive and nonprogressive) was significantly higher in the 20 μM curcumin-treated group than in the vehicle control group (*p* value ≤ 0.05) after thawing. In particular, a significant increase in spermatozoa with progressive motility was observed in the 20 μM curcumin sperm group. No significant difference in sperm motility was observed between the 2.5 μM, 5 μM and 10 μM curcumin-treated sperm groups and the control group (Table 3).

**Table 3:** Effect of curcumin concentrations (2.5, 5, 10 and 20 μM) added to freezing medium on progressive motility (PR) and total motility (progressive + nonprogressive (NP)) and viability. The values were expressed as mean ± SD. *p ≤ 0.05

Treatments	Progressive motility (PR) (%)	Total motility (PR+ NP) (%)	Viability (%)
Control (freezing medium)	28.15 ± 3.50	42.59 ± 4.45	50.57± 10.28
2.5 μM Curcumin (freezing medium + curcumin 2.5 μM)	27.78 ± 5.00	40.35 ± 2.57	51.23± 8.56
5 μM Curcumin (freezing medium + curcumin 5 μM)	26.50 ± 4.05	39.75± 5.65	49.76± 9.87
10 μM Curcumin (freezing medium + curcumin 10 μM)	35.65 ± 3.12	51.71 ± 5.36	54.78 ± 11.23
20 μM Curcumin (freezing medium + curcumin 20 μM)	47.20 ± 5.80*	65.00 ± 3.73*	56. 35 ± 12.03

### Intracellular ROS Assessment

**Fig. 1:**
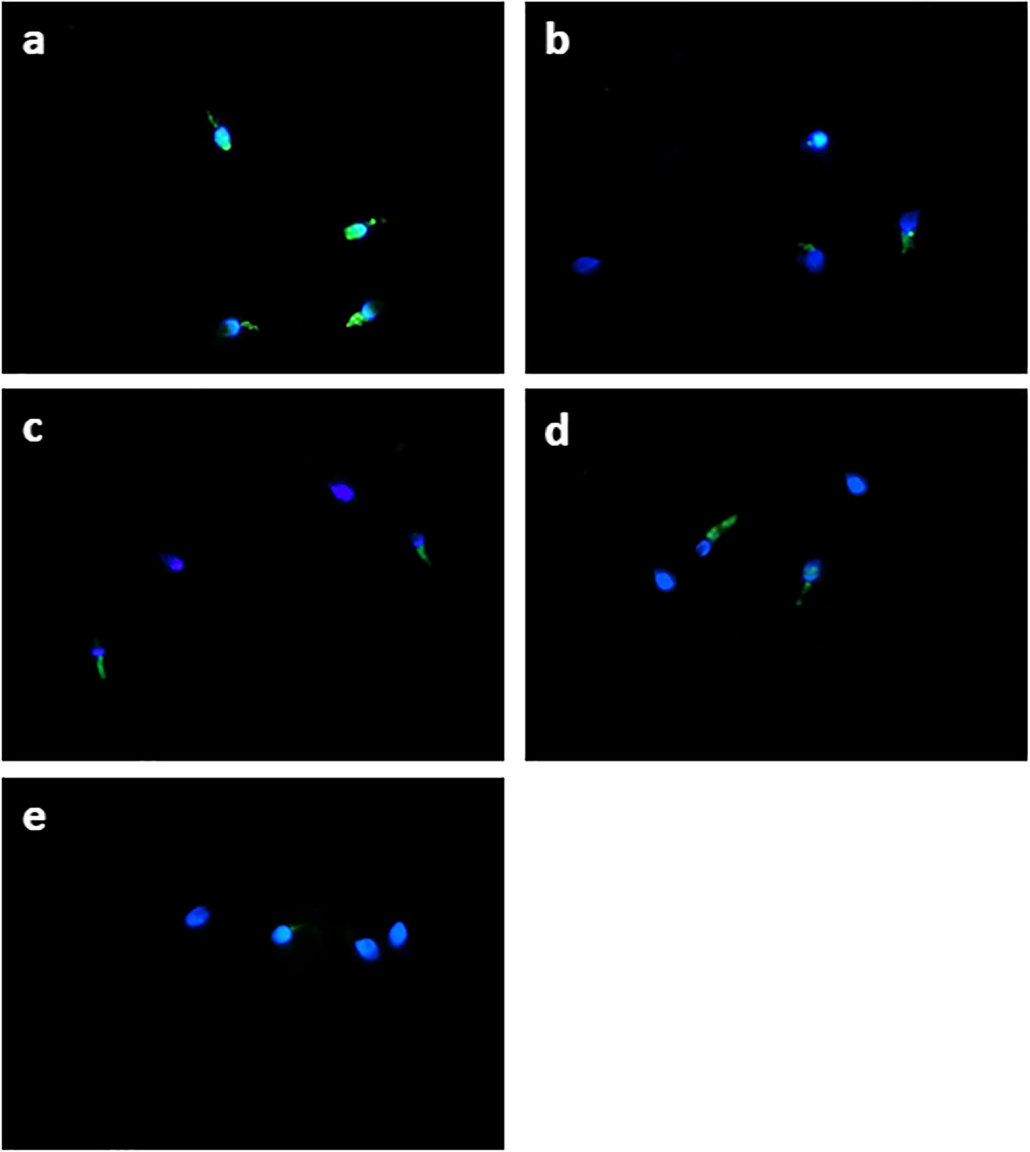
Intracellular ROS (green cell) in human sperm cells analysed by fluorescence microscopy using the DCFH_2_-DA probe in different treatment groups: **a** vehicle control; **b** 2.5 μM curcumin-treated group; **c** 5 μM curcumin-treated group; **d** 10 μM curcumin-treated group; 20 μM curcumin-treated group

**Fig. 2:**
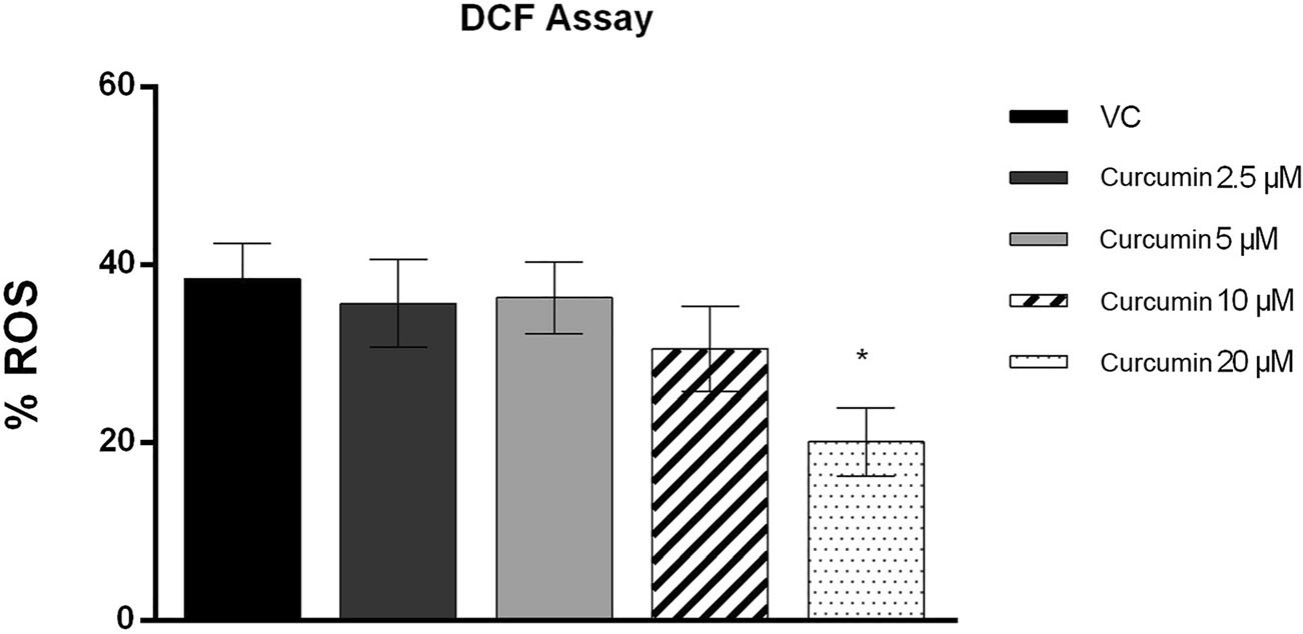
Percentage of intracellular ROS (ordinate) in human sperm cells in the different treatment groups (*n* = 12) (abscissa) after the thawing procedure. The black bars are vehicle controls (VC); the dark grey bars are the 2.5 μM curcumin-treated group (curcumin 2.5); the light grey bars are the 5 μM curcumin-treated group (Curcumin 5); the striped bars are the 10 μM curcumin-treated group (Curcumin 10); and the dotted bars are the 20 μM curcumin-treated group (Curcumin 20). The error bars represent ± standard deviation (SD). *p ≤ 0.05

The results obtained by DCF assay showed that the sperm intracellular ROS percentage was significantly lower in the groups treated with 20 μM curcumin than in the vehicle control group (*p* value ≤ 0.05). No significant difference was seen in the percentage of ROS production when the 2.5 μM, 5 μM and 10 μM curcumin-treated groups were compared with the vehicle control group (Figs. 1 and 2).

### Sperm DNA Fragmentation Evaluation

**Fig. 3:**
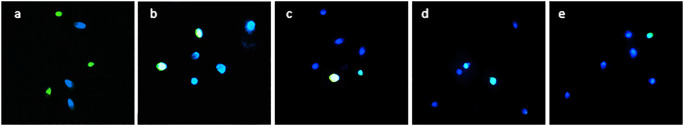
Human sperm DNA fragmentation as analysed by a TUNEL assay in the different treatment groups: **a** vehicle control; **b** 2.5 μM curcumin-treated group; **c** 5 μM curcumin-treated group; **d** 10 μM curcumin-treated group; 20 μM curcumin-treated group

**Fig. 4:**
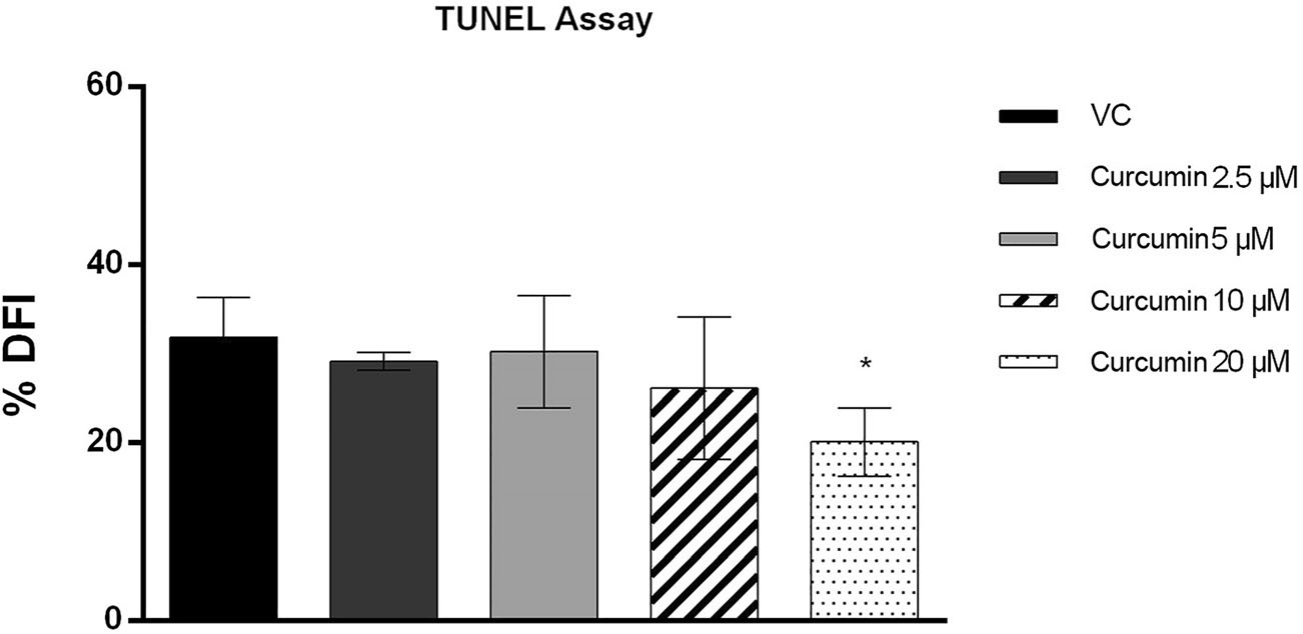
DNA fragmentation index (ordinate) in human sperm cells in different treatment groups (*n* = 12) (abscissa) after the thawing procedure. The black bars are vehicle controls (VC); the dark grey bars are the 2.5 μM curcumin-treated group (Curcumin 2.5); the light grey bars are the 5 μM curcumin-treated group (Curcumin 5); the striped bars are the 10 μM curcumin-treated group (Curcumin 10); and the dotted bars are the 20 μM curcumin-treated group (Curcumin 20). The error bars represent ± standard deviation (SD). *p ≤ 0.05

The TUNEL assay results revealed a decrease in sperm DNA fragmentation when curcumin was added to freezing medium. After thawing, the DFI was significantly lower (*p* value ≤ 0.05) in the group treated with 20 μM curcumin than in the vehicle control group. No significant difference in DFI was observed in the 2.5 μM, 5 μM and 10 μM curcumin-treated groups compared with the vehicle control groups (Figs. 3 and 4).

### Sperm *GPX4* mRNA Expression

**Fig. 5:**
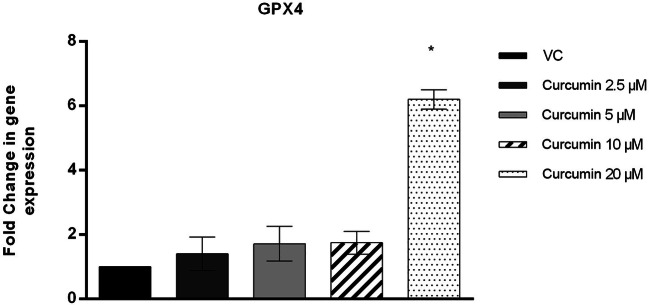
RT-qPCR analysis of *GPX*4 (ordinate) in human spermatozoa in different treatment groups (*n* = 12) (abscissa) after the thawing procedure. The black bars are vehicle controls (VC); the dark grey bars are the 2.5 μM curcumin-treated group (Curcumin 2.5); the light grey bars are the 5 μM curcumin-treated group (Curcumin 5); the striped bars are the 10 μM curcumin-treated group (Curcumin 10); and the dotted bars are the 20 μM curcumin-treated group (Curcumin 20). The error bars represent ± standard deviation (SD). *p ≤ 0.05

Sperm cells frozen with freezing medium plus 20 μM curcumin had significantly higher *GPX*4 mRNA levels than sperm cells frozen without curcumin (*p* value ≤ 0.05), whereas the *GPX*4 mRNA levels after thawing in the 2.5 μM, 5 μM and 10 μM curcumin-treated groups were not significantly different from those in the vehicle control group (Fig. 5).

## Discussion and Conclusions

Sperm cryopreservation guarantees male gamete self-conservation to preserve fertility. It is well known that these procedures impair sperm quality, reducing sperm fertilizing ability [26, 66, 67]. The major biological problem of sperm cryopreservation is due to the alteration of normal cellular mechanisms: sperm cells exposed to low temperatures undergo irreversible damage that causes decreases in motility and fertility potential [68].

The success of sperm freezing depends on internal sperm parameters and external factors such as the composition of the diluent, type and concentration of cryoprotectants, dilution rates and cooling, equilibration, freezing and thawing procedures [69]. Cryoprotectants and suitable freezing and thawing procedures protect sperm from dehydration, increased salt concentrations and thermal shock to safeguard cell membrane integrity and to optimize the osmolarity of extracellular fluids [70]*.* However, the presence of cryoprotectants is not enough to protect cells from the stress induced by the freezing process, which in most cases involves membrane integrity loss, mitochondrial damage, metabolic and functional status alterations [8, 71], lipid peroxidation and increased cytoplasmic ROS and DNA damage [72]. However, small amounts of ROS are important for sperm maturation; in fact, sperm ROS play an important role in capacitation, the acrosomal reaction, mitochondrial stabilization and motility [73].

The disruption of the balance between ROS and antioxidant scavenging could provoke oxidative stress. Human sperm are very sensitive to free oxygen radical toxicity, and the main consequence is lipid peroxidation [74]. The most dangerous products of lipid peroxidation are malondialdehyde (MDA) and 4-hydroxynonenal (4-HNE), which can cause severe protein dysfunction and DNA damage, recruiting leukocytes by chemotactic activity and inhibiting cell proliferation. Given the low efficiency of the intracellular systems of sperm gene repair, the only protective mechanism is the tight packing of the genome and the antioxidants present in the male genital tract and in the seminal plasma [75]. Nfr2 plays a critical role in the defence against oxidative stress by inducing the expression of antioxidant proteins and phase II detoxification enzymes as well as of genes encoding catalase (Cat), superoxide dismutase, glutathione S-transferase (GST) and haem oxygenase-1 [76]. Nonenzymatic and natural antioxidants also play an important role in the protection of male gametes and are able to counteract DNA damage both in vivo and in vitro [77].

Antioxidants seem to be of great clinical importance, as they reduce ROS and oxidative stress and, as a result, improve fertility potential both in natural pregnancy and in ART; they can also play a protective role during embryonic development [78].

Antioxidant supplementation can potentially improve sperm cryopreservation outcomes. The addition of 5% sericin to freezing and thawing media increased total motility and viability and decreased DNA fragmentation relative to media without sericin [79]. Melatonin may exert its cryoprotective effects on spermatozoa by counteracting intracellular ROS and thereby reducing MDA generation, leading to an increase in the post-thaw viability and motility of cryopreserved human spermatozoa [68] and reducing oxidative damage by upregulating heat shock protein 90 (HSP-90) expression [80]. Supplementation of the cryopreservation medium with quercetin induced a significant improvement in post-thaw sperm motility, viability and DNA integrity; however, it had no effect on caspase 3 activation [81].

Considering the antioxidant properties of curcumin and its capacity to improve semen quality parameters in asthenoteratospermic men by reducing oxidative stress [38, 82, 83], we evaluated the effects of curcumin (2.5 μM, 5 μM, 10 μM and 20 μM) supplementation in freezing medium on frozen-thawed human sperm cells.

Our data showed that, among the concentrations tested, 20 μM curcumin had positive effects on sperm quality after the freezing-thawing procedure compared to the controls. In particular, the protective effects of 20 μM curcumin have been highlighted as increasing sperm total motility, with the improvement of the progressive motility, and reducing intracellular ROS production, proving that this antioxidant is able to protect sperm cells from oxidative damage induced by cryopreservation and improve semen quality after thawing.

The results obtained by the TUNEL assay showed that the sperm DFI was decreased in the curcumin-treated group compared to the frozen sperm group without curcumin added to the cryopreservation medium, and 20 μM curcumin even reduced the DFI below the 26% threshold, the value above which the fertilizing capacity of fresh spermatozoa is drastically reduced [84]. Moreover, the maximum concentration of curcumin tested was able to induce increased *GPX4* gene expression and consequently enhance the cellular physiological enzyme antioxidant system and decrease sperm DNA fragmentation, thereby reducing sperm apoptotic processes. This result indicates that curcumin not only is able to directly defend sperm from ROS attack but also acts at the molecular level, enhancing the intrinsic defences of the cells.

Modulating *GPX*4 gene expression in sperm cells is a key factor in preserving male fertility [62, 63]. Gpx4 can be found in the cytosol and bound to sperm membranes or in other cells of the male genital tract [85, 86]. It is the only isoform capable of inactivating phospholipid hydroperoxides regardless of the release of fatty acids by phospholipase A2 and is therefore the only antioxidant enzyme capable of repairing some of the damage caused by radicals to macromolecules such as proteins or membrane lipids [87]. Another important function of Gpx4 is oxidation of protamine thiol groups with the formation of disulfide bridges essential for nuclear chromatin condensation and with a structural role creating a network of protein bonds typical of the capsular structure [88–90]. Therefore, in addition to its antioxidant action, Gpx4 has a key role in sperm maturation through the metabolism of hydroperoxides and oxidation of nuclear protamine [91, 92]. Moreover, the expression of Gpx4 has been correlated with the stability of sperm chromatin and with impaired spermatogenesis because it is associated with apoptotic mitochondrial pathways [93, 94].

Our results were in agreement with the findings of studies on the ameliorative effect of curcumin on the motility, viability, total antioxidant capacity (TAC) and DNA integrity of frozen-thawed rat sperm and the protective effect of curcumin on dog sperm from damage caused by cryopreservation procedures, improving sperm parameters and protecting sperm against ROS and increasing NADPH oxidase 5 (*NOX*5) gene expression [51, 95]. Similarly, in vitro supplementation of bull semen extender with curcumin was able to improve sperm motility (in particular, progressive motility) and protected the sperm from damage induced by oxidative stress [96]. We suggest that the radical scavenging activity of curcumin dose-dependently increased with increasing curcumin concentration, whereas sperm motility, ROS production, DFI and *GPX*4 gene expression were not significantly different between the control group and the groups treated with low concentrations (2.5 μM, 5 μM and 10 μM) of curcumin. On the other hand, Zhou et al. showed that curcumin might improve asthenozoospermia by reducing ROS reproduction and regulating Nrf2 levels. They also indicated that curcumin can ameliorate sperm motility in a dose-independent manner and could induce toxicity to sperm motility when applied beyond a certain concentration; 100 nM curcumin significantly increased sperm motility by reducing ROS and apoptosis, while treatment with curcumin at concentrations of 1 mM and 1 M decreased sperm total and progressive motility compared to those of the control group [41].

In any case, at certain concentrations, curcumin can improve sperm parameters due to its ability to scavenge free radicals as an antioxidant through its phenolic, β-diketone and methoxy functional groups. Curcumin can also uncouple the keap1-Nrf2 complex, which leads to Nrf2 stabilization and subsequent transport into cell nuclei, leading to the transcription of several antioxidant genes involved in antioxidant responses [97–100]. Curcumin may also influence sperm motility, capacitation and function by inhibiting tyrosine phosphorylation of sperm surface proteins and Ca^2+^ channels, acidifying the intracellular pH of sperm and hyperpolarizing the sperm cell membrane [101].

In this study, we chose to evaluate the antioxidant ability of freezing medium supplemented with low concentrations of curcumin to counteract the oxidative damage to sperm induced by cryopreservation; our results showed that the only concentration tested that was sufficient to produce a positive effect on human sperm cells after thawing was 20 μM. In fact, supplementation of 20 μM curcumin to sperm freezing medium could improve sperm parameters and decrease oxidative damage, preserving sperm DNA fragmentation by suppressing ROS production and *GPX*4 gene overexpression. Further studies should clarify the pathways influenced by curcumin supplementation during sperm cryopreservation through the modulation of the expression of other genes involved in oxidative stress, such as SOD and CAT, and by evaluating the expression of apoptotic markers such as Fas and p53. Although other groups have demonstrated the harmful effect of curcumin at higher concentrations (1 mM) [41], future prospects will be aimed at evaluating the effects of curcumin concentrations between 20 μM and 1 mM in sperm freezing medium.

Preserving sperm DNA integrity in the presence of the oxidative stress produced by cryopreservation procedures is important for fertilization outcomes, such as normal development of the embryo, foetus and infant [102–104].

Our findings are of great importance for improving the efficiency of sperm cryopreservation to help infertile/subfertile men and suggest that antioxidants, in particular curcumin, acting at different levels can restore reproductive capacities in individuals with from oxidative stress-associated dysfunction.

## Data Availability

Not applicable.
